# Emotions and emotion up-regulation during the COVID-19 pandemic in Germany

**DOI:** 10.1371/journal.pone.0262283

**Published:** 2022-01-07

**Authors:** Iris Schelhorn, Swantje Schlüter, Kerstin Paintner, Youssef Shiban, Ricardo Lugo, Marie Meyer, Stefan Sütterlin

**Affiliations:** 1 Department of Experimental Psychology, University of Regensburg, Regensburg, Germany; 2 Department of Clinical Psychology; PFH–Private University of Applied Sciences, Göttingen, Germany; 3 Faculty of Health and Welfare Sciences, Østfold University College, Halden, Norway; University of Hradec Kralove: Univerzita Hradec Kralove, CZECH REPUBLIC

## Abstract

In stressful situations such as the COVID-19-pandemic, unpleasant emotions are expected to increase while pleasant emotions will likely decrease. Little is known about the role cognitive appraisals, information management, and upregulating pleasant emotions can play to support emotion regulation in a pandemic. In an online survey (N = 1682), we investigated predictors of changes in pleasant and unpleasant emotions in a German sample (aged 18–88 years) shortly after the first restrictions were imposed. Crisis self-efficacy and felt restriction were predictors of changes in unpleasant emotions and joy alike. The application of emotion up-regulation strategies was weakly associated with changes in joy. Among the different upregulation strategies, only “savouring the moment” predicted changes in joy. Our study informs future research perspectives assessing the role of upregulating pleasant emotions under challenging circumstances.

## Introduction

By now, the COVID-19 pandemic and its consequences have reached the status of a global health crisis that is accompanied by great danger, difficulties, and doubts and characterized by a substantial amount of uncontrollability [1]. Widespread disease outbreaks comparable to the COVID-19 pandemic always showed detrimental effects on physical and psychological well-being [2]. In line with this, since the beginning of the COVID-19 pandemic, several studies showed a worrying increase in depression and anxiety disorders [3,4], general distress and sleep disorders [5,6], as well as a deterioration of already preexisting symptoms of posttraumatic stress disorder and depression [7] and eating disorders [8]. Therefore, positive and negative affective states—defined here as the superordinate categories for valenced states including emotions, emotional episodes, moods, and dispositional states [9]—that people experienced in response to the COVID-19 pandemic are of relevance. Studies demonstrated that the intensity and valence of emotional responses to negative events predict people’s successive wellbeing [10], also during the COVID-19 pandemic [11]. Consequently, affect and emotion regulation strategies applied to alter the duration or intensity of an emotional response [12] might also have had an influence on people’s affective outcomes during COVID-19. However, the following questions remain widely unanswered: Were there changes in emotional experience shortly after the COVID-19 pandemic came into existence? How did the context of COVID-19 influence the relationship between emotional responses? What are the specific predictors of changes in emotional responses during COVID-19 and which emotion regulation strategies buffered these responses?.

### Emotional responses during the early weeks of COVID-19

Previous research investigating emotional outcomes during the first weeks of restrictions in Poland reported high levels of positive affect (happiness, relaxation) and low levels of negative affect (sadness, anger, anxiety) [13], which is counterintuitive at first. The authors explained their surprising results with an unrealistic optimism bias [14], the phenomenon that individuals rate their personal risk of experiencing a negative event as below average. In line with this bias, researchers already found that people assess their own risk of contracting COVID-19 and suffering heavy consequences as below the risk of others [15]. Furthermore, the authors also discussed the creation of a sense of solidarity in society and leisure, as well as hope that the fight against the pandemic would be efficient due to the heavy restrictions in Poland, as possible causes for an increase in pleasant emotions. A longitudinal diary study that was conducted in Serbia showed a decrease in negative emotions (worry, fear, boredom, anger) over five weeks with the biggest difference in worry, followed by fear and boredom [16]. Previous studies also showed that many people evaluated COVID-19 as a threat to their emotional well-being [17] as well as describing it as provoking fear due to increasing death rates [18]. Lack of peer contact, an increase in domestic violence, unemployment, and reduced opportunities to relieve stress can add additional emotional pressure [19]. By now, documented increases in depression rates [3] and exacerbations of various other psychopathological symptoms [20] can confirm that the COVID-19 pandemic had consequences on long-term affective outcome.

### The relationship between positive and negative affective states during COVID-19

Regarding the relationship between positive and negative affective states, a negative context such as the COVID-19 pandemic might have an impact on their association: Researchers discovered that the relationship between the two superordinate affective states depends on the negative or positive valence of the context [1,21,22]. Researchers showed that in a positive context, pleasant and unpleasant emotions were not associated, whereas the occurrence of negative events caused an increase in negative affect as well as a decrease in positive affect [21]. Based on these findings, our colleague [22] developed an integrative model–the Dynamic Model of Affect (DMA) proposing that people show context dependent significant differences in their capacity to process information. The model states that highly negative events do not only elicit negative affect but evoke a stressful context typically perceived as uncertainty or threat. In this condition, information must be processed efficiently and with a clear priority to mitigate the negative effects resulting in simplicity of information processing. The overall result is typically a strong increase in unpleasant emotions along with a decline in pleasant emotions [23,24]. On the occasion of positive events, people show the capability to register pleasant emotions, which does not change the capability to simultaneously be aware of negatively rated aspects. Assuming that the first weeks after COVID-19 related restrictions were perceived as a negative event for many people, this would mean that an inverse association between changes in negative and positive affect would be expected.

### Predictors of emotional responses during COVID-19

Emotional responses during COVID-19 were significantly influenced by specific pandemic-related behavior. In general, people who tended to inform themselves intensely about COVID-19 in the media and adhered to the pandemic-related safety-measures were more worried [25]. Although younger people reported experiencing less fear and worry than older people, they reported higher levels of boredom but this could be moderated by having structured routines to decrease boredom and negative emotions [16]. Another study found conflicting results [17]. In this study, younger age was associated with increased concerns about the threat of COVID-19, higher negative affect, and lower positive affect. Younger people reported fewer positive events and lower perceived coping efficacy while older adults seemed to endure stressors more successfully and showed better emotional well-being. Young adults seemed to be particularly sensitive to specific events as they reacted with a higher increase in negative emotions when a COVID-19 related stressor occurred and a bigger decrease in these emotions in case of a positive event [17]. A transnational study during the early phase of the virus outbreak showed that the majority of participants rated these measures imposed by the governments as very helpful because they reduced the anticipated risk posed by COVID-19 [26]. Thus, the inherently uncontrollable situation could take on the appearance of controllability and positive consequences such as being able to spend more time pursuing new hobbies. This might have minimized negative affect in the early stages of the pandemic.

Appraisals such as perceived controllability are often more predictive of emotional outcome than mere circumstances [9]. Therefore, a likely predictor of affective outcome in a crisis is self-efficacy. Perceived self-efficacy is defined as the ‘beliefs in one’s capabilities to organize and execute the courses of action required to produce given attainments’ [27, p. 3] and is divided into a specific and a global component. General self-efficacy relates to the overall belief that one is in control over one’s own life, actions, and decisions, while specific self-efficacy is the belief into one’s performance in a situation. General self-efficacy beliefs are a reliable predictor of behavioral intentions in various contexts and predict self-efficacy in the specific context of a crisis such as a public health disease threat [1]. Crisis self- efficacy (CSE) beliefs according to [28] include a person’s beliefs that “*s/he can successfully perform behaviors to reduce the possibility of damage in the pre‐crisis stage*, *to perform the required response activity in the initial event stage*, *and to maintain performance of the response activity throughout the maintenance stage*” (p.3). CSE can be moderated by age, being a parent, and higher income. Participants with high (CSE) experience mastery during a crisis and consequently, more pleasant emotions than those lower in CSE [29].

Regarding the effect of emotion regulation during COVID-19, previous studies have mostly focused on downregulation of unpleasant emotions while overlooking the upregulation of pleasant emotions [30]. It is therefore unclear whether emotion up-regulation strategies such as those suggested by [31,32] can help people maintain pleasant emotions or might buffer the experience of unpleasant emotions during a pandemic. According to Gross’ process model of emotion regulation [12,31], emotion generation, intensity, and duration can be influenced by situation selection, situation modification, attention deployment, cognitive change, and response modulation. Other researchers further subdivided these categories: For instance [33], lists four response modulation strategies upregulating pleasant emotions: Being present, behavioral display, capitalizing, and positive mental time travel. With regard to crises [34], proposed that resilient people may recover more quickly from negative events because they use pleasant emotions to cope with the stressful situation. Also, longitudinal evidence indicates that people struggling with bereavement show better long-term adjustment when being able to experience and express pleasant emotions in a non-loss context [35]. This suggests that pleasant emotions may be adaptive irrespective of contexts, whereas the adaptiveness of unpleasant emotions is context-bound. Emotion up-regulation during a pandemic has, to our knowledge, never been investigated.

**Fig 1:**
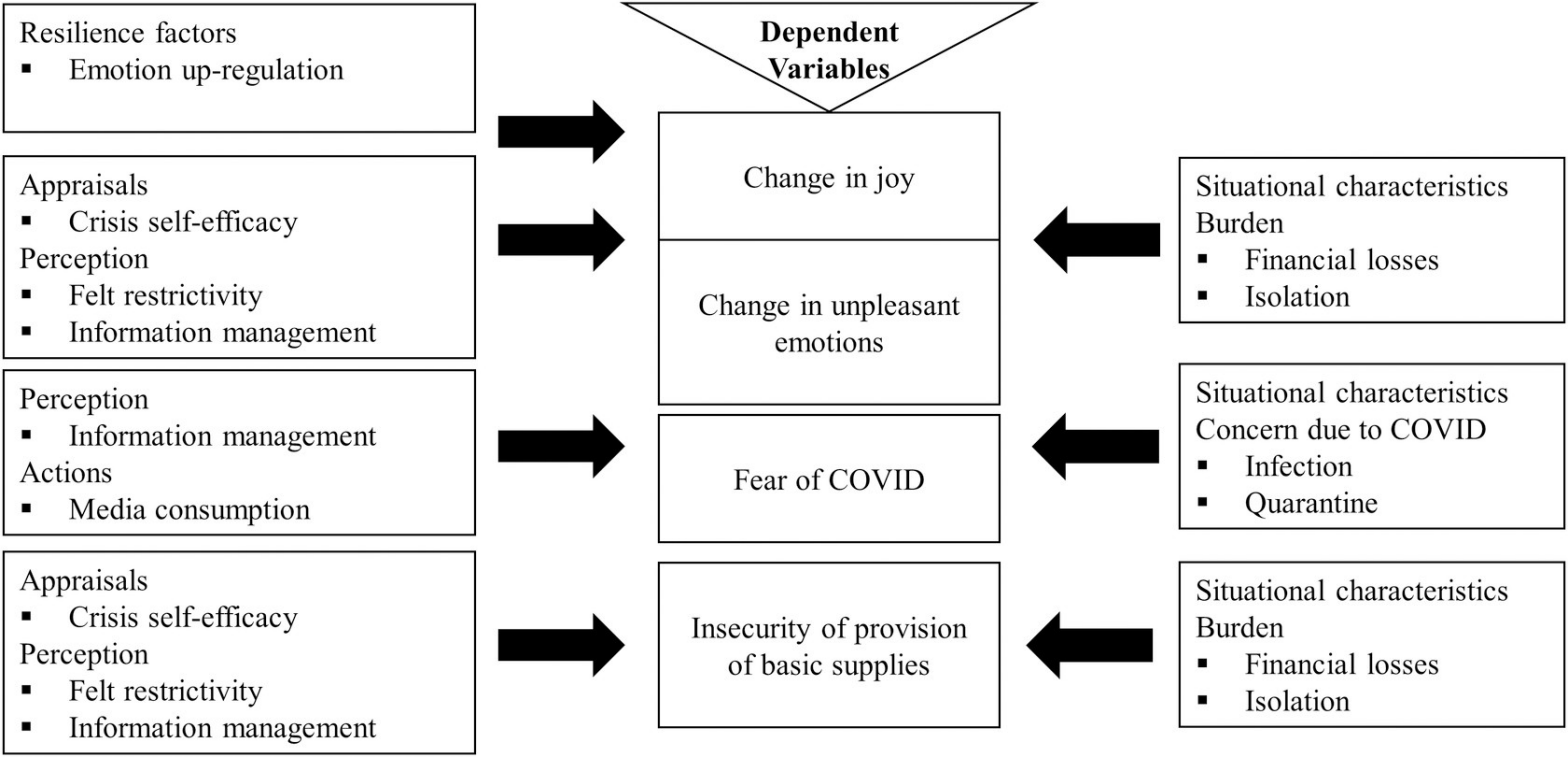
Overview of predictors included in the regression models.

In this study, we aimed at examining associations between changes in pleasant and unpleasant emotions following the outbreak of COVID-19 and subsequent restrictions in Germany, expecting an inverse relationship between changes in pleasant and unpleasant emotions (H_1_). Our second goal was to investigate predictors of changes in affective states, reported fear of COVID, and feelings of insecurity of provision of basic supplies. We collected data predicting self-reported changes in levels of joy, several unpleasant emotions, as well as data predicting fear of COVID and feelings of insecurity of provision of basic supplies in reaction to heavy restrictions that were imposed during the first months of COVID-19 crisis in Germany. Age was included in all models as it is a known predictor of both positive and negative affective outcome [36]. All specific predictors can be seen in Fig 1.

For the prediction of changes in joy and unpleasant emotions, we additionally expected the actual and perceived burden through restrictions and environmental changes as well as the perception of information management and crisis-self-efficacy to be predictive of an incline in unpleasant emotions (H_2_) and a decline in joy (H_3a_). For joy as an outcome variable, we also expected emotion up-regulation strategies to be an important resilience factor (H_3b_). For COVID-related fear, we expected personal concern, exposure to COVID-related media content, and information management by governments to be important predictors of fear level (H_4_). For feelings of insecurity of provision of basic supplies, actual droppings in financial income, household size, exposure to COVID-related media content, and clarity of media content provided by governments were included as predictors into the model (H_5_).

## Materials and method

### Participants

**Fig 2:**
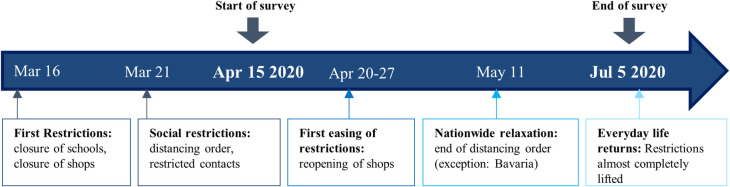
Survey and restriction timeline.

Participants of this online questionnaire based cross-sectional study were recruited via email, online social platforms, and flyers as part of a larger study project. The study was approved by the Ethics Committee of the Department of Psychology at the PFH—Private Hochschule Göttingen (Ethics application number: 251982). The patients/participants provided their written informed consent to participate in this study. Data were collected from April 15 to July 5 2020 using a 20-minute online survey in response to the official declaration of COVID-19 as a pandemic on March 11 2020, mainly in the regions of Bavaria and Lower Saxony in Germany. On March 21, the heavy restrictions were imposed (see Fig 2). A total of 1741 individuals participated in the study. Participants who were younger than 18 years or did not live in Germany were excluded (*n* = 59). The final sample consisted of 1682 participants. Participants ranged in age from 18–88 years (*M* = 34.61, *SD* = 14.33). Demographic data are displayed in Table 1.

**Table 1:** Demographics of responders (N = 1682).

Variable	*n* (%)
Total	1682 (100%)
Gender	
Male	451 (26.8%)
Female	1224 (72.8%)
Other	2 (0.2%)
Marital Status	
Single	1017 (60.5%)
Married	549 (32.6%)
Widowed	25 (1.5%)
Separated	91 (5.4%)
Living Situation	
Alone	262 (15.6%)
Shared Flat	193 (11.5%)
With partner	499 (29.7%)
With family	728 (43.3%)
Job Status during lockdown	
Attending work as usual	447 (26.6%)
Attending work in home-office	530 (31.5%)
Mixture	396 (23.5%)
Not working	158 (9.4%)
Question does not apply to situation	151 (9.0%)
Working in the health sector	
In direct contact with COVID patients	61 (3.6%)
Not in direct contact with COVID patients	79 (4.7%)
Health worker, but not a doctor, MTA or nurse	200 (11.9%)
No	1342 (79.8%)
Education obtained	
Middle school	113 (6.7%)
High school	582 (34.6%)
Apprenticeship	308 (18.3%)
University or higher ^X^	675 (40.1%)
Else	3 (0.2%)
COVID-19 diagnosis	11 (0.7%)
COVID-19 diagnosis in friends/family	509 (30.3%)
Quarantined	108 (6.4%)

^*X*^ Postgraduate degree; Master’s degree, PhD, MD.

### Measures

#### Affective outcome—Composite score unpleasant emotions

Participants’ reported changes in unpleasant emotions since March 11. That day was chosen as a reference point because it was the day when COVID-19 was declared a pandemic. Participants rated the following four emotions: Frustration, boredom, loneliness, and isolation. The selection of unpleasant emotions was based on findings from a review of the psychological impact of quarantine from earlier epidemic outbreaks like SARS or Ebola and the studies it quotes [37]. The authors observed that quarantine measures especially led to frustration, boredom, and feelings of isolation. Participants were asked whether they agreed to the statement “I feel this emotion to a greater extent since the classification of the COVID-19 disease as a pandemic (11/03/2020) and the subsequent measures” on a 5-point Likert scale (-2 = *I disagree* to 2 = *I agree*). These four unpleasant emotions were merged into a composite score and showed acceptable reliability (Cronbach’*s* α = .70).

#### Affective outcome—Fear

Two major threats that were associated with the pandemic are the death threat or threat of physical damage by the virus itself [17] and the threat of a crisis to financial stability and safety [18]. Therefore, two questions targeting these two fears were included: Participants were asked whether they were afraid of a Sars-CoV-2-infection which could be answered on a 5-point-Likert-scale (1 = *not at all* and 5 = *extremely*). We also asked participants whether they felt that their provision with basic supplies was safe (1 = *no*, 5 = *yes*).

#### Affective outcome—Pleasant emotions

For measuring pleasant affective outcome, a single item scale was used measuring the change in self-reported joy. Participants were asked whether they agreed to the statement “I feel this emotion to a greater extent since the classification of the COVID-19 disease as a pandemic (11/03/2020) and the subsequent measures” on a 5-point Likert scale (-2 = *I disagree* to 2 = *I agree*).

#### Upregulation of pleasant emotions

Literature still lacks a self-report questionnaire measuring both antecedent- and response-focused emotion up-regulation strategies. Therefore, for antecedent-focused emotion regulation, we generated items according to the proposed structure of the process model of emotion regulation [12] and for response-focused emotion regulation, we adapted the strategy classifications of savoring strategies by our colleagues [32,38]. Participants were asked to rate the extent to which they had used each of the following strategies since the outbreak of the pandemic on a 5-point Likert scale (0 = *does not apply*, 4 = *extremely applies*): “*I intentionally seek situations that will evoke pleasant emotions*” (Situation Selection). “*I modify situations in a way so that I can enjoy them”* (Situation Modification). “*I focus my mind on things that will evoke pleasant emotions*” (Attentional Deployment). “*I take a perspective on situations that will evoke pleasant emotions*” (Cognitive Change). “*I express my pleasant emotions through facial or bodily expression”* (Behavioral Display). “*I express my pleasant emotions by sharing them with others”* (Capitalizing). “*I enhance my pleasant emotions by reminiscing about past pleasant situations or by imagining future pleasant situations*” (Positive mental Time Travel). “*I fully focus my attention on my pleasant emotions*” (Being Present). A factor analysis [39] was run revealing a KMO-index of .90 and a significant Bartlett’s test of sphericity, *χ*(28) = 8,425.52, *p* < 0.001, suggesting that the sample was suitable for a factor analysis [39]. Just one factor was extracted with a lowest factor loading of .74 (behavioral display). The scale showed good internal consistency (Cronbach’s α *=* .91).

#### Crisis self-efficacy

Four items from the Crisis Self-efficacy Index [28] reflecting its four-dimensional structure were included. We chose those four items which had the highest load on the respective factor: “*I am certain I have the ability to take necessary action to protect myself during a crisis*” for beliefs of one’s own action efficacy, “*I am able to use resources effectively during a crisis*” for preventive efficacy, “*During a crisis*, *I can achieve most of the goals that I have set for myself*” for achievement efficacy, and “*During a crisis*, *I can usually handle whatever comes my way*” for uncertainty management efficacy. Participants rated their (dis-) agreement to these statements on a 7-point Likert scale ranging from 1 (*strong disagreement*) to 7 (*strong agreement*). In line with the back translation criteria of experience of translators and fluency in languages suggested by Back-Translation for Cross-Cultural Research by our colleagues,^(4)^ the original English version was translated by a native German speaker while the back translation was conducted by the English native speaker. An independent German/English with knowledge of self-efficacy conducted a comparison of the original English version and back translated version. As a result, a common single version was developed that was appropriate for the German culture. All items were translated from the English version into the German language and showed good reliability (Cronbach’s α *=* .85).

#### Actual and perceived burden

As mentioned in previous research, many people struggled at the beginning of the pandemic with changing circumstances as changes at work, loneliness, and problems with childcare. Of these actual circumstances, we asked about the participants’ household size and the number of children at home. As we did not measure children’s age, only household size (one vs. several persons) was included in the analysis. In order to measure actual financial insecurity, two items of our survey changes in income and level of financial aid were combined into a composite score. This composite score was coded as follows: 1–4 implicated that participants indicated a decrease in income along with four possible options of financial aid: no financial aid, financial aid requested, financial aid received, unsure yet if financial aid will be required. Participants who reported no change or an increase in income were coded with a 5 or 6. Specific burden through COVID-19 was also taken into account: Participants were asked whether they had an infection or knew someone with an infection. They were also asked about their exposure to COVID-related media content (“*How much time (minutes/day) do you spend each day searching for information about COVID-19*?”). We also asked how restricted participants felt by the imposed measures on a 5-point Likert scale (0 = *not at all*, 4 = *extremely*).

#### Information management

According to our colleagues [37]^(5)^, especially clarity and transparency of communication about the severity of the infectious disease (SARS-CoV-1) influenced affective outcome, as well as duration and duration foreseeability. Other authors demonstrated congruence and consistency in communication to be crucial during the current pandemic [40] as well as prosocial appeals being superior to threat messages as they seemed to evoke greater compliance and positive emotional responses [41]. A factor analysis provided two relevant factors regarding information management about the COVID-19 pandemic. The factor *information clarity* consisted of six items with acceptable reliability (Cronbach’s α *=* .74). Participants were asked to mark on a 5-point Likert scale (1 = *yes*, 2 = *rather yes*, 3 = *neither yes nor no*, 4 = *rather no* and 5 *= no*) whether they felt like they received sufficient information about the COVID-19 virus (*Do you feel you are receiving sufficient information on COVID-19 and related developments*? and *Do you feel you receive enough information from the government*?) as well as their opinion about that information’s truthfulness (*Do you have the feeling to get false information (Fake News)*?*)*. Furthermore, they were asked if they knew where to report when noticing possible COVID-19 symptoms as well as if (all recorded on a 5-point Likert scale reaching from 1 = *yes* to 5 = *no)* and when (*before*, *as or after the measures took effect*) the responsible government explained why the measures were imposed. The values of the composite score reached from 0 (*no*) to 4 (*yes*). A high score on all items therefore implied a high level of information clarity. The second component the factor analysis provided was *communication of duration of restrictions* consisting of two items. It measured if there was a set time period in which the COVID-19 pandemic containment measures would apply (measured on a 5-point Likert scale reaching from 1 = *yes* to 5 = *no)* and whether it was declared when they would end (*no; yes*, *limited to less than two month; yes*, *limited to two months* and *yes*, *limited to more than two months*). These scores were recoded into a composite score reaching from 0 (*no*) to 4 (*yes*) as well. High scores implied that the duration of restrictions was clearly communicated. However, due to a very low reliability (Cronbach’s α *=* .27), this scale was not included in the analysis.

### Procedure

Respondents first answered demographic questions followed by an assessment of the extent by which participants had been affected by COVID-19 and the implemented restrictions. Next, they were asked to report the changes in emotions, fill out a measure of clinical symptoms (ICD-10-Symptom-Rating^(46^), and answer items concerning the use of strategies to upregulate pleasant emotions, the use of coping humor, and perceived self-efficacy in crises. Participants filled out the survey via the online survey program “lime survey” and could leave their email address for a potential follow-up survey. No personally identifiable information was collected. All participants gave their informed consent for participation and completed the questionnaires electronically. Data was collected anonymously without IP addresses or GPS tracking.

### Statistical analysis

The statistical data analyses were conducted with SPSS® (IBM SPSS Statistics, Version 25.0). The level of significance was set to *α* = .05 for all analyses. Pearson product-moment correlations were calculated for all correlations. For the main analyses, multiple regressions were performed with forced entry method in two steps entering age as a demographic variable and situational characteristics in a first step, and entering variables measuring appraisals, perceived information management, and resilience factors in a second step. One exploratory stepwise model was calculated because we had no specific hypotheses for the impact of specific upregulation strategies on joy. Due to violations of assumptions, for the models for fear of COVID and feelings of insecurity of basic supplies, bootstrapping was applied. Analyses of indirect effects were calculated with the PROCESS tool for IBM SPSS statistics developed by our colleague [42].

## Results

### Associations between changes in affective outcome

**Fig 3:**
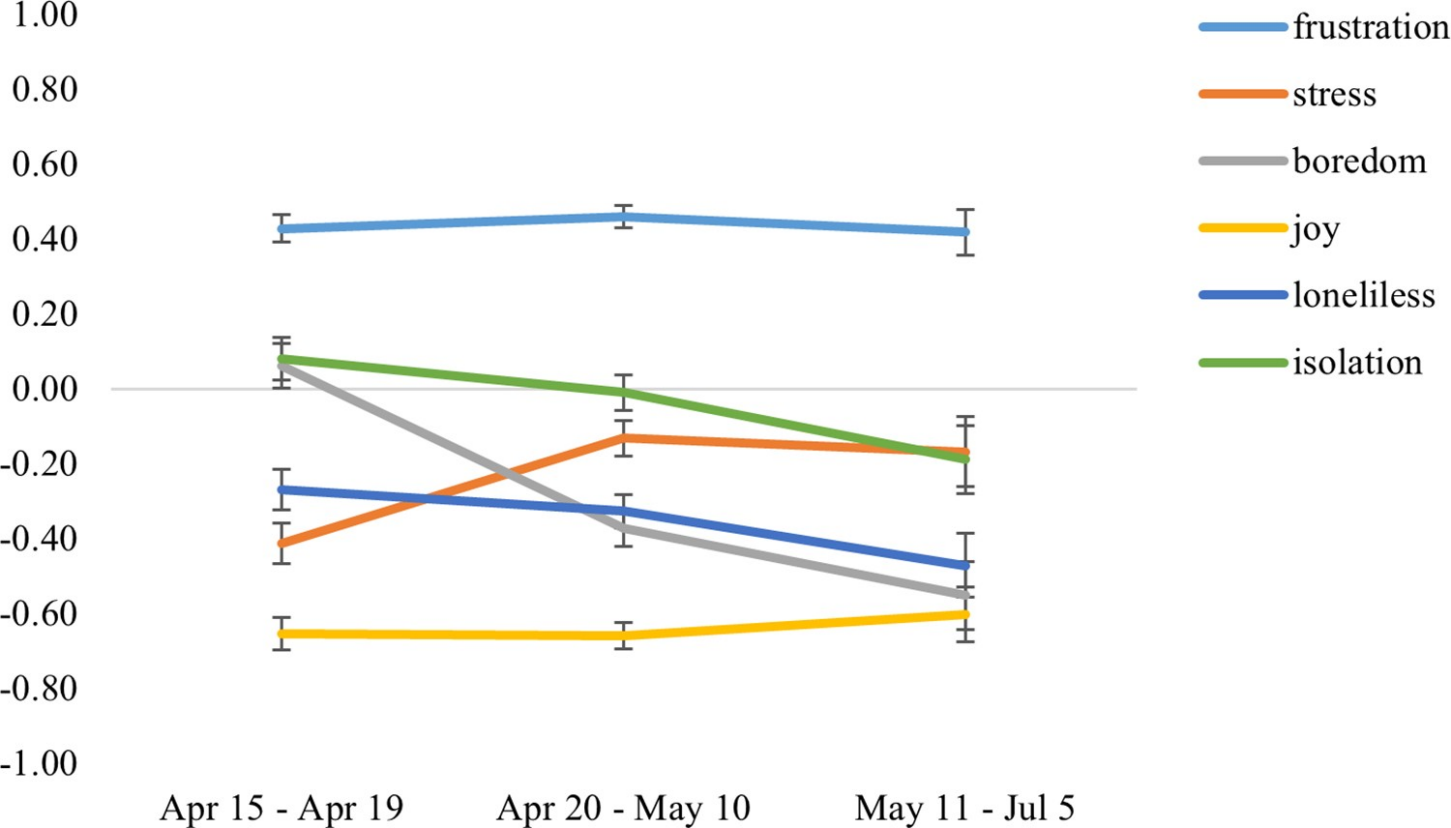
Reported changes in emotional states plotted against the timeline of restrictions.

Summing up all unpleasant emotions (boredom, frustration, loneliness, and isolation), an overall slight decrease in unpleasant feelings was observed, as can be seen in Table 2. The mean values were normally distributed. When plotted against the timeline of restrictions (Fig 3), there were significant differences over time in the reported changes in the emotional states boredom (*p* < .001), stress (*p* = .001), and isolation (*p* = .042). Compared to before 11/03/2020, participants reported an average increase in frustration, specifically, a high percentage of participants (38.2%) experienced no change in frustration, followed by 34.1% reporting a moderate increase in frustration. On the other end of the scale, no one indicated a strong decrease. We found the frustration variable to be normally distributed. Regarding feelings of isolation, in average, there was no change reported, however, the variable was negatively skewed. A closer look at the distribution revealed that the mean of *M* = 0.00 was caused by the fact that a surprisingly sizable part of the sample indicated a strong decrease in feeling isolated (22.1%). Changes in feelings of loneliness were normally distributed, and a large portion of the sample stated a strong decrease in feeling lonely (27.9%) whilst 25% of the participants reported a moderate increase in loneliness. On average, participants reported decreases in boredom and loneliness. The boredom variable was non-normally distributed with a positively skewed distribution and characterized by a high percentage of the sample reporting a strong decrease (29.1%), contrasted by relevant sample parts stating only a moderate increase in these feelings (24.9%). Concerning the emotion of joy, an average decrease of joy was observed and the variable showed a positively skewed distribution because a high percentage of the sample reported a strong decrease in joy (27.6%). Together with participants indicating a moderate decrease in joy, more than half of the participants (50.5%) experienced a decrease in joy following the pandemic outbreak. However, the largest percentage of the sample (38.2%) reported no change in joy, and more than 10% of participants stated an increase in joy. Correlational analysis to test the first hypothesis (H_11_) revealed an inverse association between changes in joy and all the negatively valenced emotions as predicted (see Table 2).

**Table 2:** Correlations between changes in unpleasant emotions and joy.

Variables	*M (SD)*	*M*_diff_(*SE*) Females-Males^X^	1	2	3	4	5	6	7
1. Joy	-0.65 (1.04)	0.09 (.06)	---						
2. Frustration	0.44 (0.90)	0.14 (.05)*	-.25** (-.29, -.20)	---					
3. Loneliness	-0.33 (1.33)	0.02 (.07)	-.22**(-.27, -.17)	.29**(.25, .33)	---				
4. Boredom	-0.25(1.43)	-0.22 (.08)*	-.10**(-.15, -.05)	.18**(.14, .23)	.38**(.33, .42)	---			
5. Isolation	0.00 (1.38)	0.02 (.07)	-.21** (-.26, -.16)	.29**(.25, .33)	.66**(.63, .70)	.36**(.32, .40)	---		
6. Unpleasant (Score)	-0.07 (0.86)	0.04 (.05)	-.29**(-.34, -.25)	.57**(.54, .60)	.78**(.76, .80)	.62**(.59, .65)	.79**(.77, .80)	---	
7. Fear of COVID	2.01 (0.98)	0.11 (.05)	-.06*(-.10, -.01)	-.00(-.05, .04)	.05*(.00, .10)	-.06*(-.10, -.01)	.03(-.01, .08)	.05(-.00, .09)	---
8. Insecurity of provision of basic supplies	4.62 (0.78)	-0.08 (.04)	.07*(.02, .12)	-.15**(-.20, -.10)	-.09**(-.14, -.04)	-.00(-.05, .05)	-.08*(-.13, -.03)	-.14**(-.19, -.10)	-.06*(-.12, -.01)

*Note*.

* = *p* < .05

** = *p* < .001 for two-tailed correlations or independent *t*-tests. Due to violations of the assumption of normal distributions, bias-corrected and accelerated bootstrap 95% CIs are reported in square brackets (Field (2013)). ^x^M_diff-_scores for males and females were calculated with *n* = 1224 females and *n* = 451 male subjects.

### Main analyses

#### Predicting changes in unpleasant emotions

To test the second hypothesis (H_22_), hierarchical regression analysis to test the effects on unpleasant emotions, age, actual (financial droppings and reception of compensation, household size) were added to the first step, and perceived burden through restrictions, perception of communication (information management), and crisis self-efficacy were added to the second step. The model explained 38% of the variance in reported change in unpleasant emotions, *F* (6, 1675) = 173.19, *p* < .001. As displayed in Table 3, the variables age, household size, information management, felt restriction, and crisis index were statistically significant predictors, with felt restriction recording the highest *β*-value followed by the crisis index. Younger, alone living, and people with uncompensated financial losses reported more negative changes in unpleasant emotions. The less restricted people felt and the better prepared to deal with the crisis, the less negative change they reported in unpleasant emotions. Financial losses lost their predictive value as soon as all the predictors were included in the model.

**Table 3:** Linear model of predictors of change in unpleasant emotions and joy.

Model	Predictors	Model for reported change in emotions since the declaration as a pandemic					
		Unpleasant emotions			Joy		
		*b*	*SE B*	*β*	*b*	*SE B*	*β*
1	Constant	0.56 (0.42, 0.71)	.08		-0.48 (-0.66, -0.30)	.09	
	Age	-0.01 (-0.02, -0.01)	.00	-.23**	-0.01 (-0.01, -0.00)	.00	-.08*
	Household size	0.27 (0.16, 0.38)	.06	.11**	-0.19 (-0.33, -0.05)	.01	-.07*
	Finances	-0.05 (-0.08, -0.02)	.01	-.09**	0.02 (-0.02, 0.05)	.02	.03
	Corrected *R^2^*	.07**			.01**		
2	Constant	0.56 (0.31, 0.83)	.13		-0.43 (-0.79, -0.07)	.18	
	Age	-0.01 (-0.01, -0.01)	.00	-.19**	-0.01 (-0.01, -0.00)	.00	-.10**
	Household size	0.19 (0.10, 0.28)	.05	.08**	-0.13 (-0.26, 0.00)	.07	-.05*
	Finances	-0.00 (-0.02, 0.02)	.01	-.00	-0.02 (-0.05, 0.02)	.02	-.02
	Information management	-0.09 (-0.13, -0.04)	.02	-.08**	-0.02 (-0.08, 0.05)	.03	-.01
	Felt restriction	0.38 (0.34, 0.41)	.02	.45**	-0.30 (-0.35, -0.25)	.02	-.30**
	Crisis index	-0.18 (-0.21, -0.14)	.02	-.22**	0.15 (0.10, 0.19)	.02	.16**
	Emotion up-regulation	---	---	---	0.07 (0.02, 0.13)	.03	.06*
	Corrected *R^2^*	.38**			.14**		
	*ΔR* ^*2*^	.31			.13		

*Note*. Confidence intervals are reported in parentheses. Significant *β*-values are indicated

* = *p* < .05, highly significant

** = *p* < .001.

#### Predicting changes in joy

For the regression with changes in joy as outcome variable, we used the same predictors as for the model of unpleasant emotions (H_3a3a_), in addition, the use of emotion up-regulation strategies was included in the model (H_3bb_). The complete model explained 14% of the variance in reported change in joy, *F* (7, 1674) = 38.87, *p* < .001. The five variables age, household size, felt restriction, emotion regulation, and crisis index were statistically significant. Felt restriction showed the highest *β*-value followed by the crisis index (see Table 3). Younger people, with less feelings of being restricted, higher crisis self-efficacy, and a higher use of emotion regulation strategies, and people not living alone reported stronger increases in joy. A second model was calculated including all predictors but the mean emotion up-regulation variable. Instead, an exploratory stepwise regression was carried out with all up-regulation strategies as separate predictors. Of all eight strategies included, only savoring the present moment was then included in the model, *F* (8,1673) = 35.01, *p* < .001; corrected *R*^2^ = .14. The *β*-value for savoring the present moment was .81, *p* < .001. In a further step, correlations of the eight strategies were also analyzed, revealing only highly significant and mostly high correlations between the strategies, the lowest correlation was *r* = .449, *p* < .001 for situation selection and behavioral display.

#### Predicting COVID fear

For COVID-related fear, we expected age, personal concernment (infection with Sars-Cov-2, infection of a close person, quarantine), exposure to COVID-related media content, and information management to be important predictors of fear levels (H_44_). There were no sizable correlations between predictors, *Durbin-Watson* = 2.06, all *VIF* < 1.11, all *tolerance statistics* > .90, but due to the violations of the assumptions of homoscedasticity and normal distribution of residuals, we executed the analysis with a bootstrap function for robust confidence intervals. The bootstrapped model explained 2% of the variance in reported fear of contracting the COVID-19 virus, *F* (5, 1676) = 8.51, *p* < .001. The variables age, media exposure, and information management were statistically significant, with media exposure recording the highest *β*-value. People who were younger, felt better informed, and spent more time-consuming corona-related news reported higher levels of COVID fear. When all factors were included in the model, age was no longer significant. All *β*-values and bootstrapped coefficients are depicted in Table 4.

**Table 4:** Linear model of predictors of infection with SARS-Cov-2, with 95% confidence intervals.

Model	Predictors	Model for fear of infection with SARS-Cov-2		
		*b*	*SE B*	*β*
1	Constant	1.85 (1.73, 1.98)	.07	
	Age	0.01 (0.00, 0.01)	.00	.07*
	Infection with SARS-Cov-2	0.16 (-0.57, 1.07)	.43	.01
	Infection of a close person	0.00 (-0.10, 0.10)	.05	.00
	Quarantine	-0.12 (-0.32, 0.07)	.10	-.03
	Corrected *R*^*2*^	.003*		
2	Constant	1.50 (1.29, 1.70)	.11	
	Age	0.00 (-0.00, 0.01)	.00	.03
	Infection with SARS-Cov-2	0.19 (-0.55, 1.18)	.44	.02
	Infection of a close person	-0.02 (-0.13, 0.08)	.05	-.01
	Quarantine	-0.13 (-0.33, 0.06)	.10	-.03
	Media exposure	0.00 (0.00, 0.01)	.00	.11**
	Information management	0.13 (0.06, 0.20)	.03	.10**
	Corrected *R^2^*	.02**		
	*ΔR* ^*2*^	.02		

*Note*. Coefficients were bootstrapped with bias correlated and accelerated confidence intervals.

Significant *β-values* are indicated

* = *p* < .05, highly significant

** = *p* < .001.

#### Predicting feelings of insecurity of provision of basic supplies

For fears of insecurity of provision of basic supplies, age, actual burden (financial droppings and reception of compensation, household size), exposure to COVID-related media content, information management, and the crisis index were included as predictors in the model (H_55_). We executed a bootstrap analysis because the normal distribution assumption and heteroscedasticity were violated, *Durbin-Watson* = 1.930, all *VIF* < 1.09, all *tolerance statistics* > .90. The complete model explained 18% of the variance in reported feelings of insecurity of provision of basic supplies, *F* (6, 1675) = 61.87, *p* < .001. The variables actual droppings in financial income as well as information management, and crisis self-efficacy were statistically significant, with information management recording the highest β value as can be seen in Table 5. People who had fewer financial restrictions, less droppings in financial income, felt better informed, and had higher (sic!) crisis self-efficacy reported higher feelings of insecurity of basic supplies.

**Table 5:** Linear model of predictors of feelings of insecurity of provision of basic supplies.

Model	Predictors	Model for feelings of insecurity of provision of basic supplies		
		*b*	*SE B*	*β*
1	Constant	4.10 (3.95, 4.25)	.08	
	Age	0.00 (-0.00, 0.00)	.00	-.00
	Household size	-0.04 (-0.15, 0.06)	.05	-.02
	Finances	0.13 (0.10, 0.16)	.02	.26**
	Corrected *R*^*2*^	.06**		
2	Constant	2.86 (2.58, 3.14)	.15	
	Age	-0.00 (-0.01, 0.00)	.00	-.04
	Household size	-0.04 (-0.14, 0.06)	.05	-.02
	Finances	0.10 (0.08, 0.13)	.01	.21**
	Information management	0.29 (0.23, 0.35)	.03	.28**
	Media exposure	0.00 (-0.00, 0.00)	.00	-.02
	Crisis index	0.11 (0.07, 0.15)	.02	.16**
	Corrected *R^2^*	.18**		
	*ΔR* ^*2*^	.12		

*Note*. Coefficients were bootstrapped with bias correlated and accelerated confidence intervals.

Significant *β-values* are indicated

* = *p* < .05, highly significant

** = *p* < .001.

#### Indirect effects on changes in unpleasant emotions and joy

The analyses of indirect effects for age on changes in unpleasant emotions revealed significant mediation effects through information management, *b* = -.001, 95% BCa CI [-.001, -.001] and crisis self-efficacy, *b* = -.002, 95% BCa CI [-.003, -.001]. Obviously, these effects were all extremely small. Further analysis revealed two rather small moderation effects for age by felt restriction, *b* = -.003, 95% BCa CI [-.006, -.001], *p* = .008 and age by information management, *b* = -.006, 95% BCa CI [-.010, -.002], *p* = .002. Simple slope analysis revealed that the relationship between age and unpleasant emotions was always negative, however, this association became the more pronounced, the more restricted and the better-informed participants felt. Regarding the drop in financial income, corresponding with the results of the regression analysis, its effect on changes in unpleasant emotions was fully mediated through information management, *b* = -.007, 95% BCa CI [-.011, -.030], felt restriction, *b* = -.031, 95% BCa CI [-.044, -.019], and crisis self-efficacy, *b* = -.013, 95% BCa CI [-.021, -.006]. Last, the effects of household size on changes in unpleasant emotions were mediated by felt restriction, *b* = .052, 95% BCa CI [.004, .100], and moderated through a household size by crisis self-efficacy interaction, *b* = .102, 95% BCa CI [.017, .187], *p* = .019. Simple slope analysis revealed that the positive association between low household size and negative changes in unpleasant emotions only existed, when crisis self-efficacy was medium high or high.

Regarding changes in joy, the effect of age on joy was partially mediated through felt restriction, *b* = -.043, 95% BCa CI [-.086, -.004]. We also found moderation for the positive association between low age and positive changes in joy through a joy by felt restriction interaction, *b* = -.004, 95% BCa CI [-.007, -.001], *p* = .026. The association between low household size and negative changes in joy was mediated through crisis self-efficacy, *b* = .001, 95% BCa CI [.001, .002], and emotion upregulation, *b* = -.001, 95% BCa CI [-.001, -.000]. Both these effects were extremely small. We found moderation through a household size by information management interaction, *b* = -.206, 95% BCa CI [.394, -.019], *p* = .031. Simple slope analysis revealed that the relationship between the two variables did not exist for participants with low values in information management.

## Discussion

The aim of the present study was two-fold: First, changes in pleasant and unpleasant emotions were examined and their associations following the outbreak of COVID-19 and subsequent restrictions in Germany. Second, predictors of changes in affective states were investigated focusing on the role of upregulation strategies for changes in joy during these circumstances. Our main analyses resulted in two models with an adequate data structure and high predictive value for unpleasant emotions and joy, while models for fear of COVID-19 and insecurity of provision with basic supplies could not be sufficiently validated.

The model for unpleasant emotions had the strongest predictive value confirming the relevance of our selection of predictors. As expected, living alone and being younger predicted increases in unpleasant emotions with relatively small effects. Interestingly, the effect of changes in financial income was significant for unpleasant emotions but not for joy, and its effect on changes in unpleasant emotions was almost completely mediated through felt restriction and crisis self-efficacy. The effect of household size was partially mediated through felt restriction and crisis self-efficacy acted as a moderator. Living alone was positively associated with increases in negative emotions only then, when crisis self-efficacy was at an average or a higher level. The reason for this rather paradoxical effect could be that alone-living people high in crisis self-efficacy had higher action tendencies and a higher sense of controllability at first. They then possibly had to realize at a certain point that in their situation any means of stopping loneliness, isolation, or boredom had been taken from them resulting in more unpleasant emotions than for people who had declared their situation as uncontrollable from the beginning. Lastly, both changes in unpleasant emotions and joy were strongly linked to crisis self-efficacy and felt restriction, indicating that appraisals of the situation instead of the situation itself were significant. This is in line with appraisal and emotion theories [43,44].

Perceived information management was also predictive of unpleasant emotions, people who experienced the information management as more informative and sufficient more often reported to have experienced a decrease in unpleasant emotional states. On the other hand, perceived information management was positively associated with fear and feelings of insecurity, which is in line with comparable results showing that a high level of information about the virus predicted symptoms of generalized anxiety [45]. These results implicate that finding a balance between providing citizens with sufficient information but not scaring them at the same time would be the best way to prevent rising levels of unpleasant emotions during the ongoing COVID-19 pandemic as well as possible future crises.

For the model predicting joy, perceived levels of restriction, household size, and age were significant negative, crisis self-efficacy and emotion regulation were significant positive predictors. With regard to age, younger people rather reported increases in joy. Thus, it appears that younger people reported slightly stronger increases in both unpleasant emotions and joy. This might indicate that the environmental changes had a rather emotionally destabilizing effect on younger people. The effect of age on changes in unpleasant emotions was partially mediated through felt restriction. Furthermore, people living alone rather reported slight decreases in joy, which seems only natural as other possibilities of social contact were limited. This association was only found in participants that perceived the information management as rather better, supposedly, because they then rather believed what they heard and anticipated possible negative future consequences for alone-living people. The selection of emotion up-regulation strategies according to the model by our colleague [31] significantly predicted changes in joy but with small effects. The inclusion of each separate emotion regulation strategy in the stepwise model revealed that savoring the present moment was the strongest predictor; however, the intercorrelations between all the strategies were extremely high. A factor structure separating antecedent and response-focused strategies as suggested by the model could not be detected. This can be seen as slightly complementing the considerations of prior research, where the process model of emotion regulation was criticized because the existence of these two factors has never been validated [46]^(33^. Within the use of individual strategies, savoring the moment explained the largest amount of variance in the regression analysis. Similar findings on the elevated role of savoring the moment were provided by a study from our colleagues [47]^(30)^. In the study, the authors found savoring the moment to be the only upregulation strategy that yielded higher pleasant emotions during negatively valenced events. In another study [48], savoring the moment was shown to be successful for upregulating pleasant emotions in the context of few occurrences of positive events. In their study, the positive outcome of savoring the moment on pleasant emotions was equally high for groups reporting either a high or a low number of positive events. These findings suggest that savoring the moment might be especially powerful in challenging times.

Our two models for Fear of COVID-19 and insecurity of provision of basic supplies had to struggle with methodological issues because they probably represent as dependent variables only part of what they were meant to measure and therefore lack construct validity. Still, from an exploratory perspective, some conclusions can be drawn. First, in the model for COVID-fear, the finding is replicated that exposure to COVID-related media content leads to an increase in fear levels [16]. Furthermore, feeling better informed also led to elevated fear of COVID-19. The impact of age was negligible. In the model for provision of basic supplies, which still had a reasonable fit after bootstrapping, it was interesting to see that perceived information management was highly predictive for reported feelings of insecurity in the way that when people felt well informed, they felt more insecurity. A similar effect was found with state of financial income, the less droppings people actually had, the more insecure they felt. Again, crisis self-efficacy had a reasonable predictive value.

In terms of the appearance, nature, and associations of change in emotional states, we found validation for the results of our colleagues [13]^(32)^ who found a low frequency of unpleasant emotions compared to pleasant emotions, as our sample reported a decrease in unpleasant emotions. Within the various types of unpleasant emotions, only frustration showed an increase in our sample. Isolation remained unchanged whereas the remaining emotions (boredom, loneliness) showed slight decreases. However, over time, when some restrictions were lifted participants reported to feel less isolated. In line with the predictions of the DMA [22], both emotion types were inversely correlated in our study. This could indicate that in a pandemic either stability or increases in perceived joy have the effect of buffering an increase in negative affective states or limiting vulnerability to increases in negative affect. Therefore, an overall experienced increase in unpleasant emotions did not happen in the first weeks of COVID-19 and its restrictions in Germany. One potential explanation for the findings may be that three of the evaluated unpleasant emotions (frustration, boredom, isolation) were derived from a review about the impact of strict quarantine measures in previous disease outbreaks [37]. As the German government imposed rather soft quarantine restrictions, unpleasant emotions may not have been as affected as reported by other researchers [37]^(5)^. As reported in another study about the ramifications of the restriction measures [49], other unpleasant emotions may have been more affected by COVID-19 and its restrictions, such as anxiety, anger, and sadness. The study by our colleagues [13]^(32)^ examining the emotional ramifications of COVID-19 and the associated health measures in Poland found these specific unpleasant emotions to be dominant and elevated. This is partially replicated in our study because we found an increase in frustration. When it comes to joy, the DMA suggested a general decrease when facing stressful circumstances. In principle, our data support this theory because we observed a small mean decrease in joy. Interestingly, our colleagues [13]^(32)^ found that their sample reported high frequencies of pleasant emotions such as happiness and relaxation. However, the data of our sample was collected at a later point in time, which might be crucial because the authors hypothesized that an increase in joy at the beginning of the crisis could be linked to the belief that the virus could be managed and overcome, a hypothesis which is supported by our results showing that crisis self-efficacy predicted changes in joy.

This study is limited in scope because we used a gender-imbalanced German convenience sample, and its results need to be considered in light of several limitations. First, when assessing the relationship between pleasant and unpleasant emotions, our colleagues [50]^(50)^ suggested a strong dependency on the overall context, however, it remains unclear whether our sample perceived COVID-19 and the restrictions as a negative or stressful event. We can only say that it elevated self-reported frustration levels. Furthermore, our sample only included German participants, which is why we cannot generalize these findings to other cultures. This is important to note because people with different cultural backgrounds differ in their preferences for positive emotions and therefore their regulation [51]. Secondly, retrospective self-assessment of emotions was used and can be prone to biases in memory recollection at the specific time point and situation when the survey was administered. Another study [52] showed that extraverted people, for instance, tend to report more experiences of pleasant emotions in a retrospective view compared to momentary emotion ratings. Thus, variations in emotion ratings can also be reflections of personality differences. Moreover, our assessment timeframe was relatively broad, so that participants rating their emotions in April might have faced different circumstances regarding COVID-19 and associated restrictions than participants who filled out the questionnaires towards the end of June when some of the restrictions had already been lifted and infection cases had come down. Another limitation can be seen in the fact that we only assessed one pleasant emotion (joy). Complimentary pleasant emotions like gratitude, contentment, and love could have been helpful to get a more detailed view of the overall level of pleasant emotions. These emotions have been reported to occur in previous crises like the 9/11 terrorist attacks [53]. Furthermore, we used a non-validated instrument for measuring self-reported emotion up-regulation strategies. Last, while we successfully chose important predictors for changes in unpleasant emotions, we did not include relevant predictors in our model for joy like specific behavior that helped people remain resilient or joyful during the first weeks of the pandemic. For example, other studies have shown that physical activity during quarantine or lockdown led to higher positive affect [54], correlated with lower stress levels [55], and lower negative affect [23]. The discovery of new hobbies, engaging in sports or gardening, as well as childcare led to positive affective outcomes [56].

Cognitive appraisals such as crisis self-efficacy as well as emotion up-regulation strategies had beneficial effects in the first weeks of restrictions during the COVID-19 pandemic. They should therefore be highlighted in future epidemic situations both for research and for implementing interventions targeted at mental health issues or general weariness in a long-term negative event like the COVID-19 pandemic. Possible intervention measures could focus on increasing the perceived value of everyday situations since that elevates positive emotions [32]. Specifically, practicing being present could be a valuable method to upregulate positive emotions during the current or future crises. Those interventions should be targeted at young adults because they reported an increase in both negative and positive emotion induced by the pandemic, indicating that they might be more susceptible to emotional change during a pandemic. The appraisal of restrictiveness thereby seemed to have a very specific and strong effect. This study highlights that psychological factors such as objective and clear information appraisals, crises self-efficacy, and emotion up-regulation can positively influence how people feel in a long-term negative situation. Future studies should also include a focus on positive as well as negative affects’ influence on mental health outcomes when investigating long-term negative experiences to further highlight how the two processes influence behavior.

## Supporting information

S1 File Analysis of violations of assumptions for regressions. (DOCX)
