# Supplementary material for: Emotions and emotion up-regulation during the COVID-19 pandemic in Germany
Source: PLoS One. 2022 Jan 7;17(1):e0262283. doi: 10.1371/journal.pone.0262283 (PMC8741032; doi:10.1371/journal.pone.0262283)
Supplement: S1 File — (DOCX) [file pone.0262283.s001.docx]

**Tests for violations of assumptions for regression analysis:**


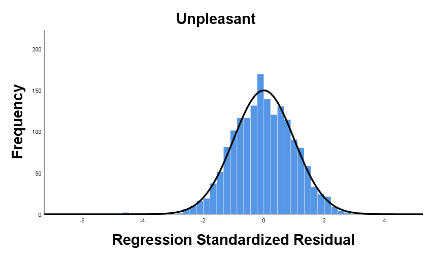

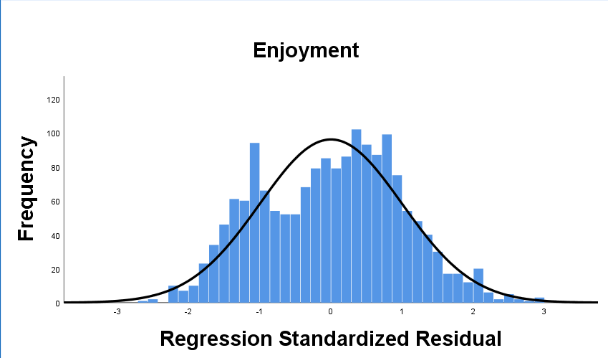


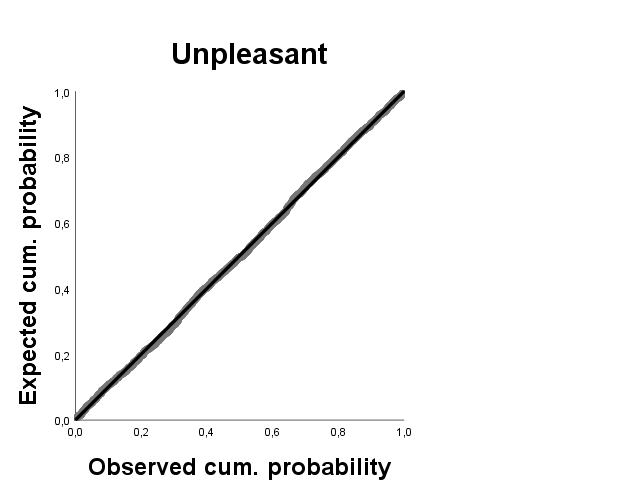

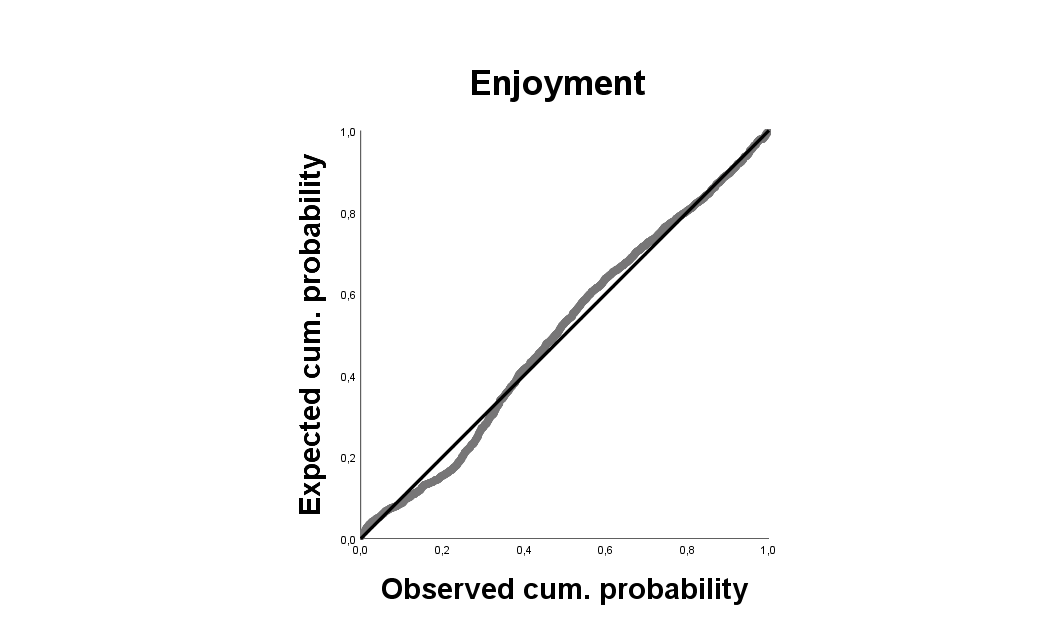


**Joy**:

There were no notable correlations between predictors, *Durbin-Watson* =1.957, all *VIF* < 1.192, all *tolerance statistics* > .839 and no signs of non-linearity or heteroscedasticity.

**Unpleasant**:

There were no substantial correlations between predictors, *Durbin-Watson* = 2.016, all *VIF* < 1.191, all *tolerance statistics* > .840 and no signs of non-linearity or heteroscedasticity.

**Fear of COVID and insecurity regarding provision of basic supplies:**


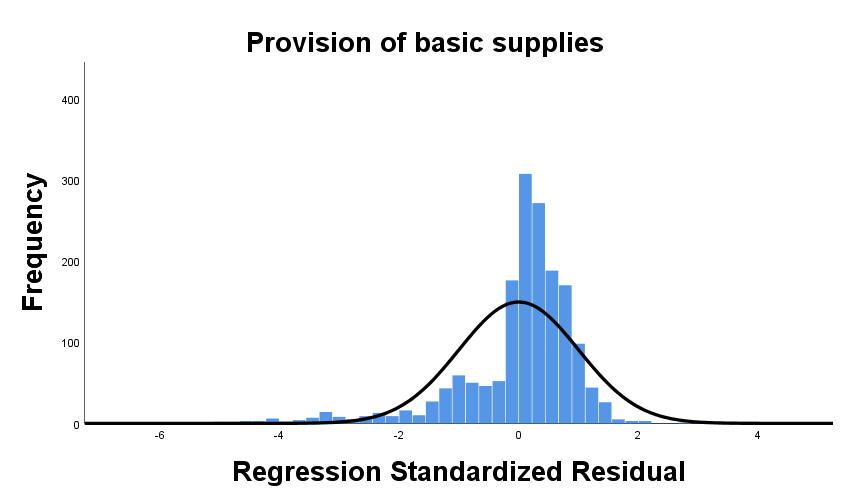

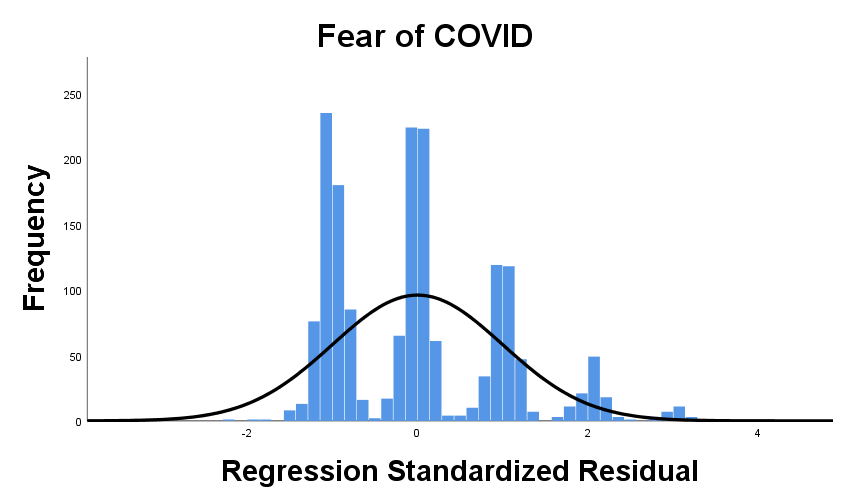


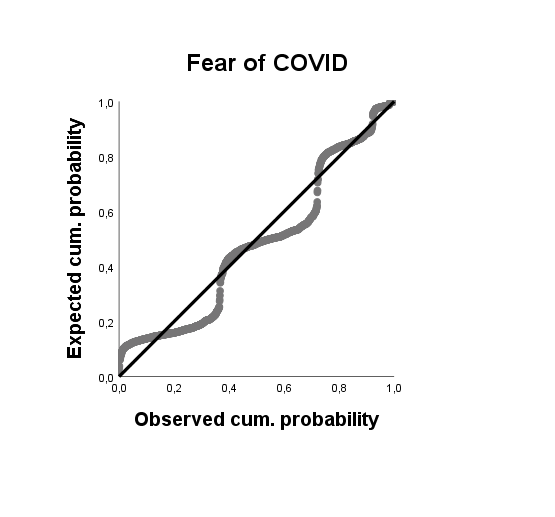

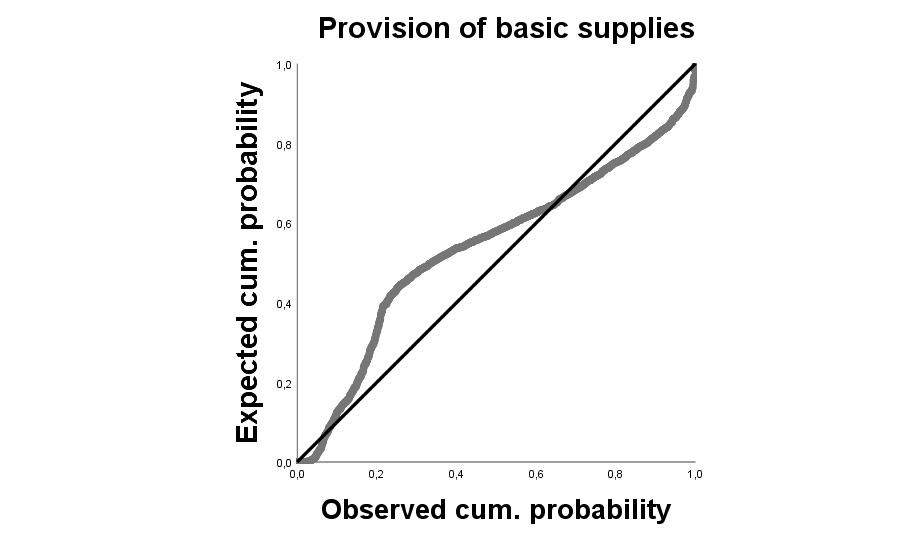


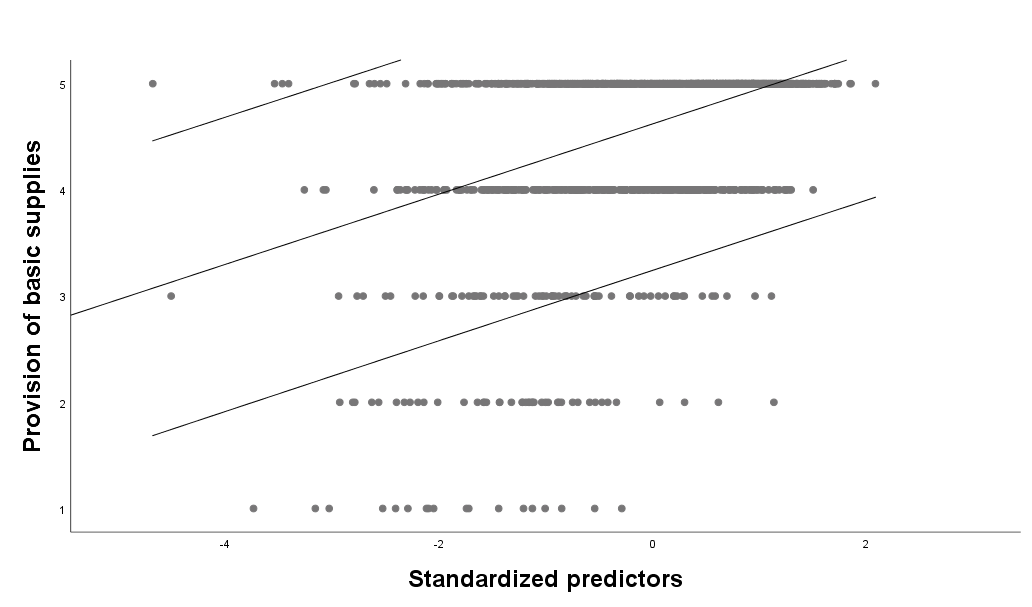
**
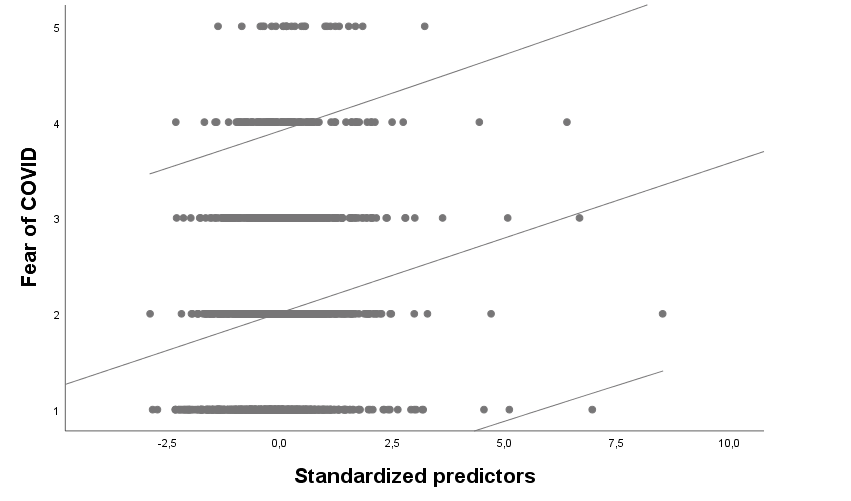

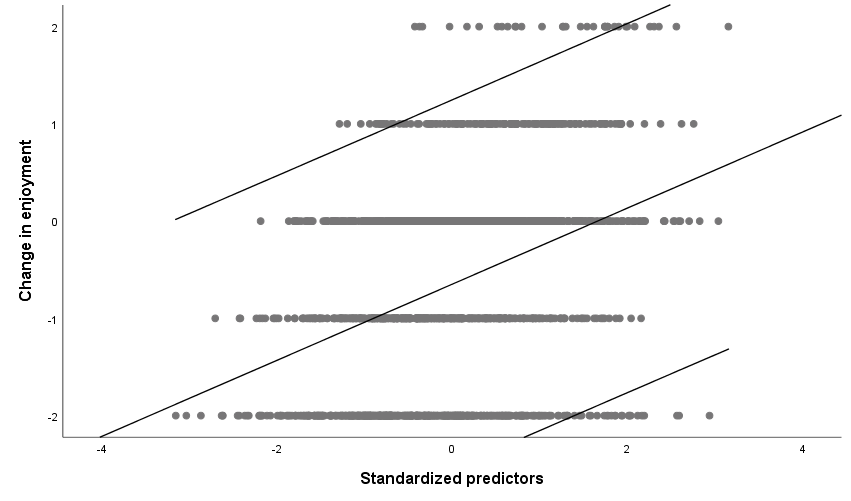

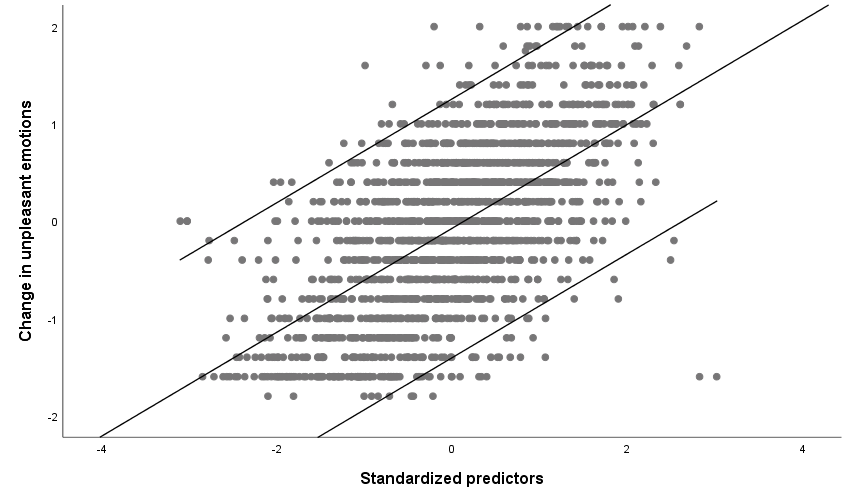
**

Standardized predictors regressed against change in unpleasant emotions (above left), change in enjoyment (above, right), fear of COVID (below, left), provision of basic supplies (below, right) and 95% -confidence intervals.
